# Filling Roots with Chloro-Percha

**Published:** 1894-03

**Authors:** R. I. Blakeman

**Affiliations:** New York.


ARTICLE VII.

### FILLING ROOTS WITH CHLORO-PERCHA.

BY DR. R. I. BLAKEMAN, NEW YORK.

This solution, depending on the degree of fluidity, is very permeative. Perhaps some of you may have experienced getting it on your fingers; if so, you have doubtless noticed how it penetrates the fissures of the skin, and how difficult it is to remove at the time without the aid of a solvent. If an instrument be dipped into it, especially one that is a little rough, the gutta-percha adheres to it very closely, and remains so after the chloroform has evaporated. It sticks to the smooth surface of glass as well, and also to tooth-structure. It answers nicely to line gold with against which amalgam is to be placed, so that the gold may not be affected by the mercury. When the fluid is very thin, it seems as susceptible to capillary attraction as water. Therefore, as some of the canals we wish to fill are very fine, and we feel that they must be filled, as on this largely depends the future welfare of the tooth, to fill them with this solution seems practical so far as the principles of physics are concerned. For the purpose of describing the process of manipulation, let us consider a molar, the roots of which must be filled from a posterior cavity difficult of access, a somewhat common occurrence. When the roots are dry and ready to fill, it is best to add some fresh chloroform to the solution kept on hand, so that the upper portion is quite thin, while the lower is left very thick. Then with a small broach, with a few fibers of cotton wrapped about the end, the solution can be carried to the canals, and, when the entrance to them is flooded over, it can be jumped in with a small bare broach.

After the canals are full of the thin solution, by dipping deeper into the supply the thicker gutta-percha is obtained, which can be pumped into the canals in like manner, the chloroform being worked out so that it can be evaporated with the chip-blower. If there should be a doubt as to the fluid having gone to the apex of any canal, it can be pushed further by making a piston of warm gutta-percha. But great care should be taken in doing this, and the patient should be instructed to respond to the first sensation, for sufficient force may be brought to bear unconsciously to push the fluid through the foramen. When the canals are sufficiently large to permit of it, it is best to put in a gutta-percha point after they are full of the solution, but not so tight as to cause pressure at the end of the root. It might be well to emphasize this point, as any one not accustomed to filling roots in this way is liable to force something through the apical foramen.—*International.*

